# Characterization of novel bacteriophage PSKP16 and its therapeutic potential against β-lactamase and biofilm producer strain of K2-Hypervirulent *Klebsiella pneumoniae* pneumonia infection in mice model

**DOI:** 10.1186/s12866-023-02979-7

**Published:** 2023-08-23

**Authors:** Sara Rahimi, Mehdi Bakht, Amir Javadi, Farshad Foroughi, Seyed Mahmoud Amin Marashi, Farhad Nikkhahi

**Affiliations:** 1 Medical Microbiology Research Center, Qazvin University of Medical Sciences, Qazvin, Iran; 2 Student Research Committee, Qazvin University of Medical Sciences, Qazvin, Iran; 3 Department of Community Medicine, Qazvin University of Medical Sciences, Qazvin, Iran; 4 Department of Immunology, School of Medicine, Qazvin University of Medical Sciences, Qazvin, Iran

**Keywords:** Bacteriophage, Hypervirulent, *Klebsiella pneumoniae*, β-lactamase, Biofilm, Mice model

## Abstract

**Background:**

Severe infections caused by β- lactamase producers, hypervirulent *Klebsiella pneumoniae* (BhvKp) with K2 serotype, highlight emergency need for new therapeutic strategies against this pathogen. We aimed to assess the efficacy of a novel phage, PSKP16, in the treating of pneumonia induced by BhvKp in mice models.

**Method:**

Genome sequences of PSKP16 were analyzed, and associated information can be found in NCBI. We applied treatment in two ways: by using mice for immediate and delayed treatments. Moreover, acute pneumonia obtained by BhvKp with intranasal method, was characterized in terms of histopathology of pulmonary lesions, biomarkers of inflammation level, leukocytes cells infiltration extent in mice, and was assessed treatment of them with PSKP16 multiplicity of infection (MOI: 10), either individually or in combination with gentamicin. Assessment of the ability of PSKP16 to inhibit BhvKp biofilm was studied.

**Results:**

PSKP16 was associated with the *Drexlerviridae* family, and had a genome size of 46,712 bp, and 67 predicted ORFs. Herein, prompt phage administration’s efficacy to decrease bacterial load and improve the survival rate in pneumonia models was faster than the synergism model with delay, but both almost displayed similar endpoints. The distribution of BhvKp strains in the lung was consistent with the histopathological findings, simultaneous inflammation, and level of serum tumor necrosis factor-α (TNF α). The phage treatment presented a lack of severe lesions and alveolar edema, reduction of inflammatory cell infiltration, which not only was it not associated with an over-inflammation but also provided a faster correction of blood cell count abnormalities compared to gentamicin. Phage with a high concentration in in vitro model effectively eliminated biofilms.

**Conclusion:**

It is essential to raise clinical awareness and management of BhvKp infections, signaled as the next superbug in waiting. The results of our study underscore the importance of PSKP16 as a phage with promising therapeutic potential in treating BhvKp-induced pneumonia.

**Supplementary Information:**

The online version contains supplementary material available at 10.1186/s12866-023-02979-7.

## Introduction

*Klebsiella pneumoniae* (*K. pneumonia*) as an encapsulated bacillus colonizes on the human mucosal surfaces including the gastrointestinal tract and oropharynx, where it can be a source of serious infection of the urinary and respiratory tracts, particularly in immunocompromised individuals, neonates, and the elderly. Nowadays, pneumonia caused by *K. pneumoniae* is considered one of the most common causes of hospital-acquired pneumonia in the world [1]. Encapsulated strains of many bacteria (e.g., *K. pneumoniae*) are more virulent and resistant to phagocytosis and some antibiotics than non-capsulated strains [2]. Capsular serotypes K1 and K2, which are the most frequent isolates from patients worldwide, have been identified as risk factors for liver abscess and complicated endophthalmitis [2]. One of the most resistant species that gives rise to hypervirulent clones, is *K. pneumoniae* (HvKp) with extended virulence factors. The HvKp strain was first reported in China in 1986 in a case series of individuals with meningitis or pulmonary involvement, endophthalmitis, pyogenic liver abscess and also associated with the underlying disease of diabetes mellitus [3, 4]. Over the ensuing decades, cases were reported throughout the Asian Pacific Rim, but cases have now emerged outside of this geographic region. The plasmid in HvKp isolates contains genes that result in increased capsule synthesis and increased siderophore production [5, 6], which have been established as one of the most important virulence factors for HvKp [7]. A number of known virulence genes associated with HvKp that contribute to colonization, invasion and pathogenicity include enterobactin siderophore (*entB*), aerobactin siderophore biosynthesis (*iucA*) and its captor (*iutA*), capsular polysaccharide (*magA, wcaG*, *wzy-k1*,*wzi*), hyper capsule: regulator of mucoid phenotype (*rmpA*), type 3 fimbriae (*mrkA* and *mrkD*), and type 1 fimbriae (biofilm-associated genes: *fimH*) [7].

The severe ascent in the outbreak of multidrug-resistant (MDR) and extremely drug-resistant (XDR) pathogens belonging to the Enterobacteriaceae group is a main economic problem, as these pathogens are prevalent natural residents of the human and animal microbiome [8]. Carbapenems are considered to be the most reliable last-resort treatment for bacterial infections because they are highly effective against many bacterial species and less vulnerable to most beta-lactam resistance determinants [9]. The carbapenems are safer to use than other last-line drugs such as polymyxins. For all those reasons, the advent and rapid expanse of carbapenem resistance in all continents, which are considered the last resort antibiotics for the treatment of Extended Spectrum Beta-Lactamases (ESBL)-producing *K. pneumonia*, constitutes a universal public-healthcare problem. Despite its extra clinical importance, there is still rare data on the advent of MDR/XDR and HvKp strains along with strong biofilm formation capacity [5, 6]. Last sporadic reports of carbapenem-resistant hypervirulent *K. pneumoniae* (CR-hvKP) indicated the emergence of this new superbug [10]. CR-hvKP is undoubtedly a superbug and its relative reports are increasing [11]. Thus, the occurrence and spread of ESBL or carbapenem resistance-hvKP pose a considerable threat to public health and should be monitored.

Biofilm matrix renders the extra resistance power for MDR/XDR bacteria which makes them resistant to harsh conditions and antibiotics which leads to the emergence of bad bugs infections like MDR, XDR, and totally drug-resistant bacteria [12]. These organisms or superbugs become in forms resistant to almost all clinically important antimicrobials. High mortality rates and extended hospitalization coupled with high costs are often associated with infections caused by this organism. The lack of progress in developing new antibacterial agents has greatly revived interest in using phages as a natural enemy of bacteria, and as a therapy to conflict antibiotic-resistant [13]. Lytic bacteriophages replicate within a particular bacterial host and cause rapid lysis and cell death within a short period [14]. Bacteriophage therapy is regaining attention as a potential treatment option for bacterial infections, including against superbugs [15]. Isolation and characterization of lytic bacteriophages against MDR/XDR- *K. pneumoniae* with a specific activity of bacteriophage have been reported in several studies [16]. Virulent phages may produce extracellular enzymes, such as depolymerase, capable of degrading the biofilm EPS matrix. In addition, bacteriophages do not cause an undesirable immune response [17]. In the present work, a novel phage (PSKP16) that specifically infects Beta-lactamase-producing and hypervirulent *K. pneumonia* strain (BhvKp) possessing the K2 serotype was isolated. Besides, the genetic background of PSKP16 was defined by bioinformatics analysis. Its therapeutic efficacy was evaluated in a murine nasal infection model.

## Results

### Bacterial strains and phage

The properties of all of the 30 investigated *K. pneumoniae* strains in this work are listed in Table 1. In our study, five capsular types (K1, K2, K5, K20, and K54) were identified among the 30 isolates. Most of the isolates were K1/K2 serotypes. Phage PSKP16 infected four out of 30 K*. pneumoniae* isolates, which had similar capsular serotype (K2). Tracing the lineage of four strains was carried out by MLST, which were belonged to the three different sequence types –ST (ST3500, ST273, and 2 cases of ST2558). In this study, although the HvKP strains possess more virulence genes, but they did not show any significant difference between biofilm formation and positive String-test compared with carbapenemase-producing cKP strains with K1/K2 serotype (Table 1). Using Bhvkp isolate, we isolated and characterized two phages native to the hospital sewage. When PSKP16 was cultured with this strain, the phage formed clear plaques and was surrounded by a large halo in plaque assay. As the most active phage against one of the isolates of CR/ESBL-hypervirulent K2-*K. pneumonia*, was selected for further characterization.

**Table 1:** The properties of thirty *ESBL /CRKP K. pneumonia* strains used in the study

**Isolat es**	**ESBL genotype**	**Carbapenemase** **genotype**	**MIC (mg/L)** **Colistin**	**MIC (mg/L)** **Tigecycline**	**MDR/XDR**	**Biofilm**	**String-test**	**Capsule serotype**	**CPS biosynthesis**			**Adhesion**		**siderophores**				
									*rmpA*	*magA*	*wcaG*	*mrkD*	*fimH*	*entB*	*iroB*	*iutA*	*iucA*	*ybt*S
KP1	SHV, CTX-M, TEM	OXA-48	2	0.5	MDR	Intermediate	+	K54			+	+	+	+				
KP2	SHV, CTX-M, TEM		0.5	0.5	MDR	Strong	+	K1		+	+	+	+	+			+	
KP3^a^	SHV		0.5	0.25	MDR	Strong	+	K2	+			+	+	+	+	+	+	+
KP4	SHV, CTX-M, TEM		16	0.5	XDR	Strong	+	K2	+			+	+	+				
KP5	SHV, CTX-M, TEM	OXA-48	0.5	0.5	XDR	Strong	+	K2				+	+	+				
KP6	SHV, CTX-M, TEM		16	0.25	MDR	Strong	+	K1			+	+	+	+				
KP7	SHV,TEM		0.5	0.5	MDR	Intermediate	-	K non-T				+	+					
KP8	SHV, CTX-M, TEM		0.5	0.5	MDR	Strong	+	K1				+	+	+				
KP9	SHV	OXA-48	0.5	0.25	MDR	Strong	+	K1	+	+		+	+	+	+	+		
KP10	SHV, CTX-M, TEM		0.5	0.5	MDR	Weak	-	K non-T				+	+	+				
KP11	SHV, TEM		0.5	0.5	XDR	Weak	-	K non-T				+	+	+				
KP1^b^	SHV, CTX-M, TEM	OXA-48	16	0.25	XDR	Strong	+	K2	+		+	+	+	+			+	
KP13	SHV, CTX-M, TEM	OXA-48	0.5	0.5	XDR	Strong	+	K1		+		+	+	+				
KP14	SHV, CTX-M, TEM	NDM-1	0.5	0.25	MDR	Intermediate	+	K2				+	+	+		+	+	
KP1^a^	SHV, CTX-M	OXA-48	0.5	0.5	XDR	Strong	+	K2				+	+	+				
KP16	SHV		0.5	0.5	MDR	Weak	-	K non-T			+	+	+	+				
KP17	SHV, TEM, CTX-M		0.5	0.25	XDR	Strong	+	K2				+	+	+				
KP18	SHV, CTX-M	OXA-48	2	0.5	MDR	Intermediate	+	K20			+	+	+	+			+	
KP19	SHV, TEM	NDM-1	0.5	0.5	MDR	Weak	-	K non-T				+	+				+	
KP20	SHV, CTX-M, TEM		0.5	0.25	MDR	Weak	-	K non-T				+	+	+			+	
KP21	SHV, CTX-M	OXA-48	8	0.25	XDR	Intermediate	+	K1				+	+	+				
KP22	SHV, CTX-M, TE	OXA-48	1	0.5	XDR	Intermediate	+	K1				+	+	+		+		
KP23	SHV, CTX-M	OXA-48	2	0.5	XDR	Strong	+	K1			+	+	+				+	
KP24	SHV, CTX-M, TEM		0.5	0.5	MDR	Weak	-	K non-T				+	+					
KP25a	SHV, CTX-M, TEM		2	0.5	XDR	Strong	+	K2			+	+	+	+		+	+	
KP26	SHV, CTX-M, TEM	OXA-48	2	0.5	XDR	Strong	+	K2			+	+	+	+				
KP27	SHV		1	0.5	MDR	Strong	+	K5			+	+	+			+	+	+
KP28	SHV,CTX-M		2	0.5	MDR	Intermediate	+	K2	+			+	+	+				
KP29	SHV,CTX-M,TEM	OXA-48	2	0.5	MDR	Strong	+	K1				+	+	+				
KP30	SHV		2	0.5	MDR	Intermediate	-	K20				+	+				+	

*CPS* Capsular polysaccharide, *K non-T* Non type able capsule

^a^Four out of 30 K*. pneumoniae* isolates that were infected by PSKP16

^b^KP12 was used in this study

In the current study, we used the BhvKp strain in the mice model for acute pneumonia infections. PSKP16 has specific features of an efficient lytic phage, it has a short multiplication time in the BhvKp, and many phages are released from the host. The one-step growth curve indicated a latent period of 10 min and a burst time of 25 min, corresponding to about 180 phage particles per infected cell.

### Morphological features of phage PSKP16 and host range determination

**Fig. 1:**
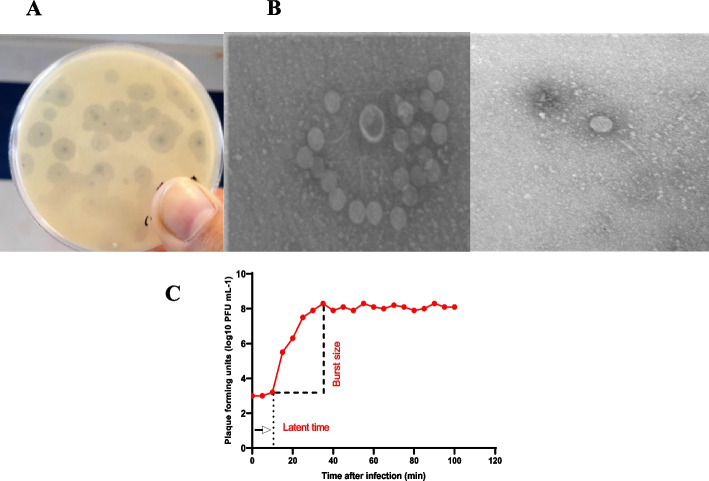
*K. pneumonia* Phage PSKP16 plaque and virion morphology on the host BhvKp. **A** the phage PSKP16 produced plaques surrounded by an expanding halo after incubation with *K. pneumoniae* strain BhvKp. **B** TEM images revealing the morphology of the phage PSKP16. Scale bars represent 100 nm. **C** One-step growth curve of phage PSKP16. A single-step growth curve of phage PSKP16 was measured against BhvKp at an MOI of 0.1. The plaque-forming unit (PFU) per infected cell at different times is shown. Data are the mean of three independent experiments

By TEM, PSKP16 phage (Gene Bank accession number OW251746.1) was identified as a virion particle that possessed an icosahedral head (60.6 ± 5 nm, *n* = 3) and approximately 150 nm long non-contractile, flexible tail. The PSKP16 phage had a typical feature of the *Webervirus* genus, the *Drexlerviridae* family (Siphovirus) (Fig. 1B). In addition, when cultured with selected BhvKp strain, the phage formed clear plaques with 1–2 mm diameter and was surrounded by a relatively large halo (3–4 mm diameter) (Fig. 1A). The host range of the phage PSKP16 was examined against various standard bacterial cultures, obtained from the microbial type culture collection. Phage did not show any lytic activity against all types of bacterial strains (ATCC) mentioned. The host range of PSKP16 was determined by spot testing on 30 K*. pneumoniae* isolates possessing known capsule serotypes (Table 1). Likewise, among the 30 K*. pneumoniae* strains that were also tested, phage PSKP16 could form both spots and plaques on four isolates including BhvKp.

The latent period of PSKP16 was 10 min, after which the number of viral particles was rapidly increased. The proliferation of PSKP16 took about 25 min to reach the growth plateau phase with a burst size of 180 PFU/cell (Fig. 1C).

### Effect of phage PSKP16 against BhvKp in invitro

To determine the lysis potency of PSKP16 in vitro against the host bacteria, the growth of the host was recorded in the presence of phage. Since the optical density (OD) of the phage-inoculated groups was much lower than the control group, phage PSKP16 is able to efficaciously lyse BhvKp in vitro (Figure S1 (Supplementary Figs. 1)). PSKP16 phage prevented the growth of BhvKp at different MOI. (The OD at 650 nm permanently increased for 16 h incubation, but it then fell down or remained stable, by different extents according to the MOI (Figure S1).

### Stability of phage particles

The results in a pH range of 3–11 showed that the phage was susceptible to PH ≤ 3 and PH ≥ 10 and was stable at a PH of 4–10 (Figure S2A). The optimal PH for this phage was 7 and 8. At PH 4 and 10, a decrease in the titer of bacteriophages was detected, while were completely inactivated at PH 3 and higher than 10. At PH 10 and 4, the number of phages reduced to 4 and 5 logs, respectively. After 1 h incubation at temperatures ranging from -20 °C, 4 °C to 50 °C, no significant reduction in phage titer was observed, whereas, at 60 °C a decrease in the titer of bacteriophages was observed. At temperatures of 70 °C and higher, phage PSKP16 was completely inactivated, and there was not any phage titer. The top activity of PSKP16 was observed at 25^ °C^ (Figure S2B). Phage PSKP16 remained active at − 20 °C for the store, as titer only slightly dropped to 10^7–6^ PFU/mL after 9 months.

### Sequence analysis and genome characterizations

**Fig. 2:**
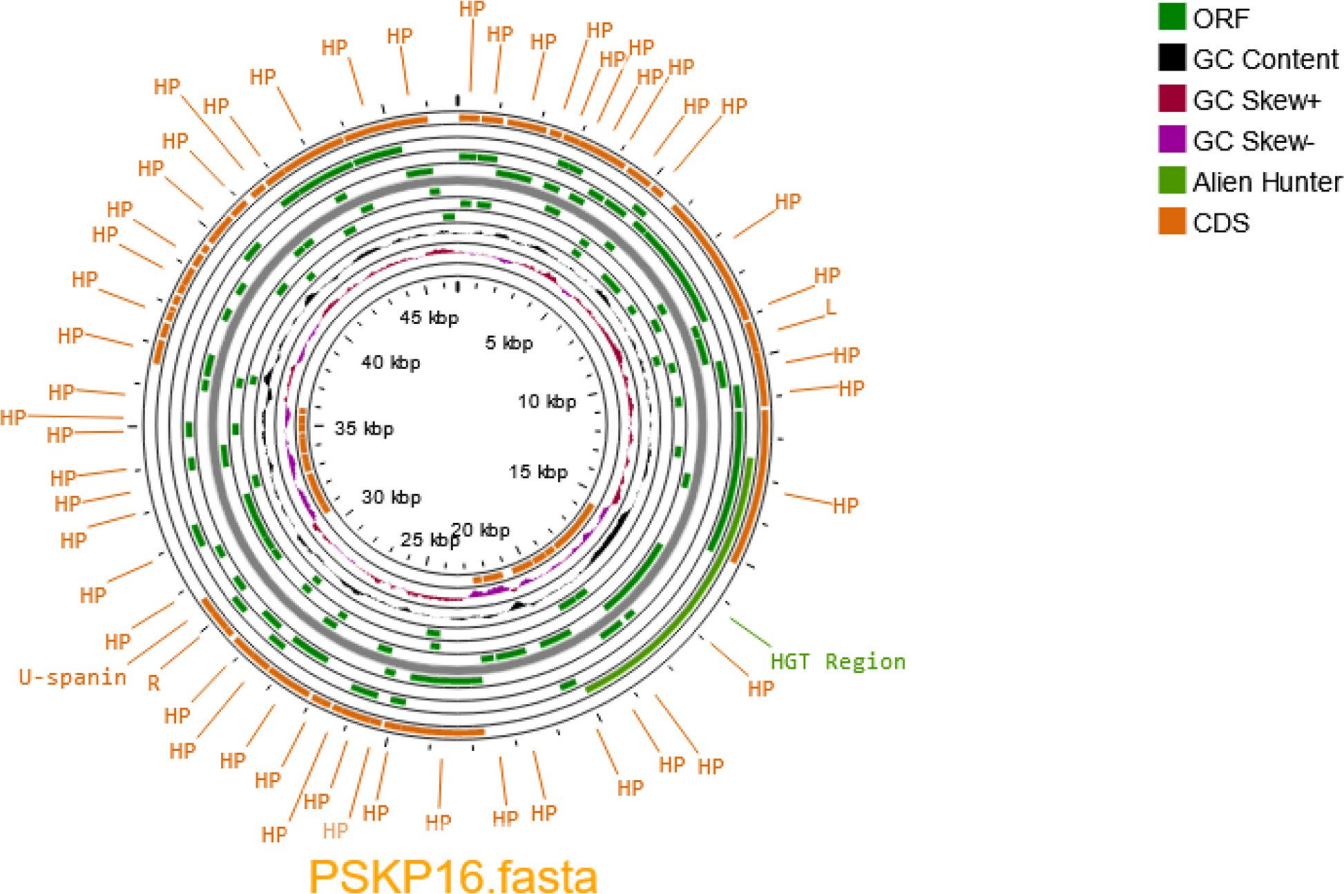
Circularized genomic ma of phage Pspk16 genome visualized using the CGView Server. Hypothetical proteins were denoted as HP. The ORFs with predicted annotations are indicated with green arrows. The inner ring shows genome location, GC skew + (red), GC skew—(purple) and GC content (black). Bioinformatics analyses of the PSKP16 genome indicated that it does not contain sequences of genes encoding integrase, recombinase, repressors and excisionase, which are the main markers

**Fig. 3:**
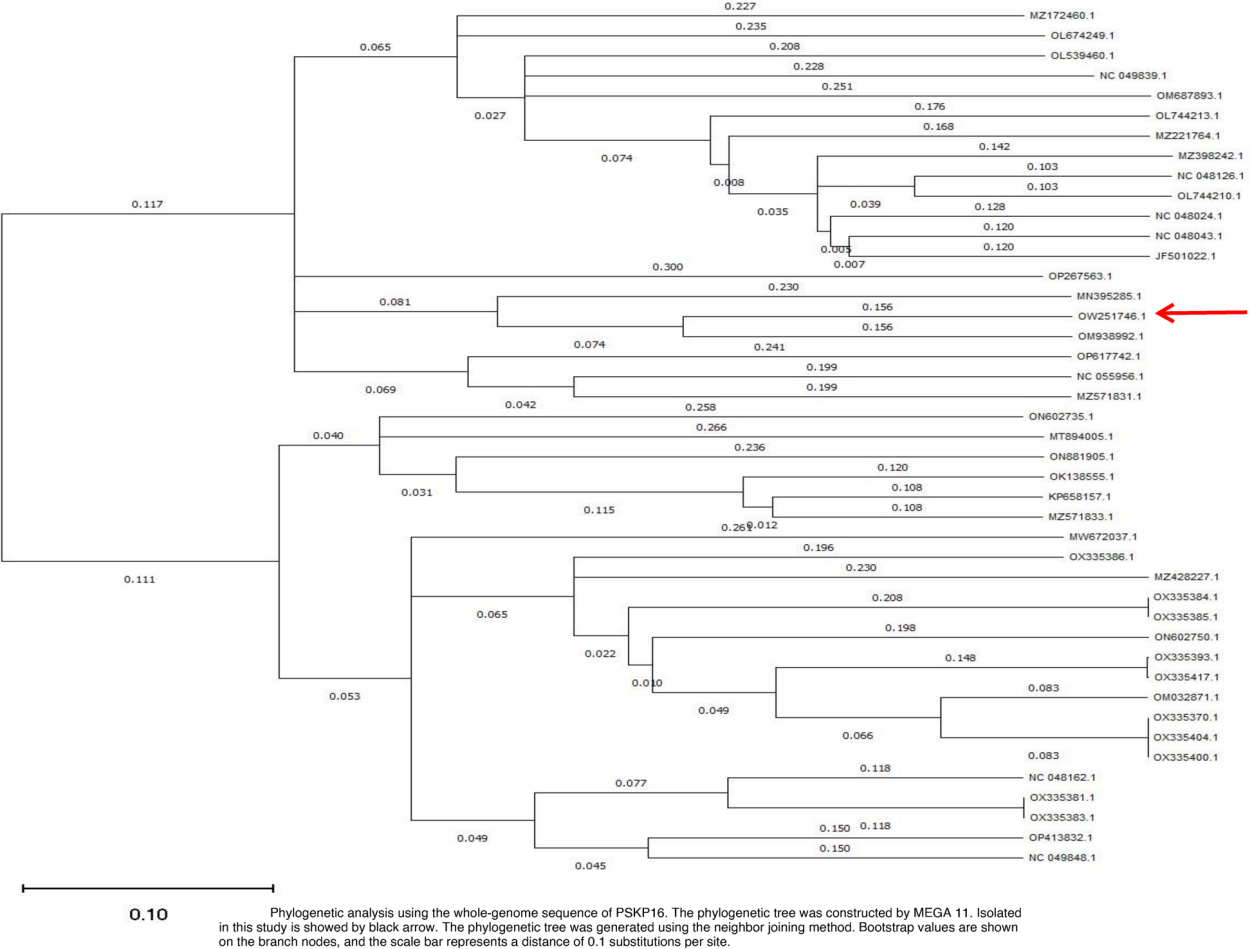
Phylogenetic analysis using the whole-genome sequence of PSKP16. The phylogenetic tree was constructed by MEGA 11. Isolated in this study is showed by black arrow. The phylogenetic tree was generated using the neighbor joining method. Bootstrap values are shown on the branch nodes, and the scale bar represents a distance of 0.1 substitutions per site

Here, we confirmed that PSKP16 belongs to the *Webervirus* genus, based on INPHARED analysis. We determined PSKP16 has a linear dsDNA genome length of 46,712 bp with a GC content of 50%. Genome annotation identified 67 ORFs, and of the coding regions, more than half were hypothetical proteins. No detectable presence of antimicrobial resistance, virulence, or integrase-coding genes was observed (Fig. 2). Genome homology analysis identified the closest related phages were B1, BMac, phi731, PWKp14, and vB_KpnS-VAC10 respectively (Fig. 3). We performed BLAST analysis (https://blast.ncbi.nlm.nih.gov/Blast.cgi) of the putative PSKP16.

### The effect of phage PSKP16 against biofilm of BhvKp strain

#### Result of colony forming unit (CFU) assay to quantify viable cells in pre-formed biofilm

The results indicated that after 24 h, the biofilm continue to form, and after 48 h, the total number of viable cells of bacterial biofilm increased. We set up MOI to remove the pre-formed biofilm, according to the phage dilution preparation from 10 to 10^8^, and quantify viable cells in pre-formed biofilm after 24 h and 48 h (Table S1). Therefore, the concentration of host bacteria (BhvKp) was determined to be 1.5 × 10^8^ CFU mL − 1 and 1.5 × 10^7^ CFU mL − 1 for biofilm formation and pre-formed biofilm, respectively. The results of the tests are reflected in Table S1.

**Fig. 4:**
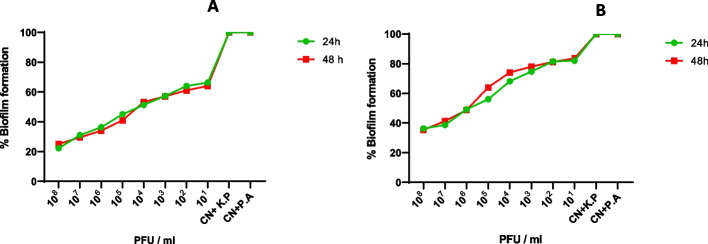
**A** Results of the effect of phage PSKP16 on biofilm formation of BhvKp strain in different MOI. **B** degradation assay of PSKP16 mediated BhvKp strain preformed biofilm in different MOI, at 24 h and 48 h old biofilm of BhvKp strain. Biofilm formation and performed biofilms grown on the microtiter plate wells for 24 or 48 h, were inoculated with 10- 10.^8^ pfu of phage PSKP16. Percentage of biofilm was scored relative to untreated control samples (100%) and represented on the Y-axes. Data represent mean ± SD from the triplicate experiments. CN + KP (Control + *K. pneumoniae* (Bhvkp)), CN + PA (Control + *Pseudomonas aeruginosa* ATCC 27853)

Biofilm formation and preformed biofilms of BhvKp were examined after co-culture and asynchronous culture using and by CV assay at two-time points (24 h and 48 h). The biofilm of BhvKp after 24 hpi was (mean OD = 3.5 ± 0.1) based on the formerly established OD cut-off (ODc) values. As well as, after 48 h (Maximum biofilm formation), the BhvKp isolate was determined as strong biofilm producer bacteria (mean OD = 4.3 ± 0.15). In this experiment, pre-established biofilm of BhvKp strain was degraded by phage PSKP16 in an MOI-dependent manner. So, significant biomass reduction during biofilm formation in the presence of PSKP16 phage (MOI 1) was reached to 75%, and 78% (*p* < 0.001) after 24 and 48 h, respectively. This value of biomass reduction in the presence of PSKP16 was continued, even if the applied phage and bacterium ratios were 1:10 (MOI 0.1), 1:100 (MOI 0.01), and less, which still led to marked biofilm degradation (Fig. 4A). To evaluate preformed and matured biofilm clearance ability, BhvKp biofilm was generated during 24 and 48 h and then treated with PSKP16 at 10^1^ to 10^8^ pfu /well. These results showed that PSKP16 could, reduce biofilms formed in vitro 18–64.6% and 16.4–63.7% at concentrations 10^1^ to 10^8^ pfu/well (p ≤ 0.001) during 24 and 48 h as measured by CV staining, compared to control, respectively. As shown in Fig. 4B, PSKP16 treatment effected an obvious reduction in biofilm biomass of BhvKp in a MOI-dependent manner, as compared with untreated controls. Data are represented as mean ± standard deviation from 3 independent experiments.

### Mice nasal colonization with BhvKp strain and phage (PSKP16) treatment model

#### Pathophysiological status at initiation of therapy

**Fig. 5:**
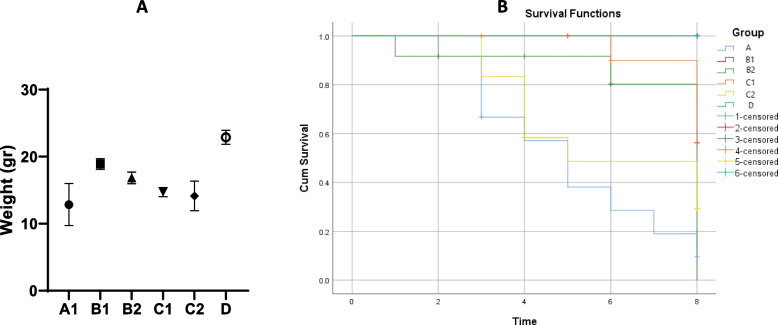
**A** Comparison the therapeutic and preventive effect of PSKP16 phage in bodyweight in a mice nasal infection model in 72 h post challenge. **B** Comparison of survival rate in 7 consecutive days post challenge. Survival rates of different groups of mice were determined. Each group contained 12 mice. Statistical analysis was performed using the Kaplan–Meier method [*P* < 0.0001, log-rank (Mantel-Cox) test]

In this study, we examined the potential usage of phage against BhvKp strain, by the PSKP16-treated mice pneumonia. Lung culture should be performed whenever possible to be registered with sustainable infection [18]. The bacterial load of BhvKp strain in the lungs of mice intranasally infected significantly increased during 26 h of monitoring, thus highlighting the ability of this strain to generate sustainable infection. To monitor the therapeutic effect of PSKP16, daily, all mice were assessed concerning their health status and categorized by surface temperature into two groups (moderate (≥ 32 °C) and severe (between 30 and 32 °C)). First, pneumonia, in less than 12 h was characterized by a concomitant decline in body temperature (30–32 °C). While group A had lower body temperature until the end of the experiment, in mice of B1,2 groups and C1,2 groups (≥ 32 °C), normal body temperature was restored gradually. In group A, those whose body temperature was between 30 and 32 died at least 3 days later. In group A, was gradually observed over 48 h post administration of BhvKp, slightly reduced motor activity, lethargy, and ruffled hair. Moderate piloerection and a change in the respiratory pattern or tachypnea were observed over 72 h post-challenge. Infected mice surviving beyond 48 h never succumbed to the infection in the next 4 days. The body weight in each group was recorded within 72 h follow-up period. The first of the decrease in body weight was created at 18 h post-challenge. Gentamicin-treated and synergism groups showed more decreased body weight and no rapid body weight recovery, respectively, because of antibiotic usage effects. Contrast, phage-treated mice demonstrated higher and more rapid body weight recovery. As far as clinical supervision is concerned, all of the mice groups showed a body weight loss characterized (except group D). Body weight were seen in 72 h post challenge by a high intra-group variation 12.8 ± 3.1 in group A and 14.15 ± 2.20 in group C2 on average ± SD, respectively. The general condition of group A, gradually deteriorated, death was seen at the end of the seventh day in eight mice. Phage-treated mice (group B1 and B2) and the mice of the group synergism (group C1) showed moderate body weight (18.915 ± 0.80, 16.83 ± 0.86, and 14.73 ± 0.68 respectively) and a clinical status characterized by marked reduced motor activity, slightly decreased respiratory rate and mild piloerection was observed. The survival rate (Fig. 5B) and health status in the B1 and C1 groups almost were similar, within 7 days' follow-up period. However, body weights in groups B1 and C1 were not identical, exactly (Fig. 5A). In group B1, mild piloerection was observed just during the first 24 h post-challenge but in other therapy groups for 3–5 days continued. Overall, the groups that received phage, while reducing their mortality rates, improved their body weight gain from the fifth day. After the fifth day, the activity and growth in these groups were regular, and hair was normal, even though they had living members after 16 days.

#### Isolation of bacteria and bacteriophage from lung tissues

**Fig. 6:**
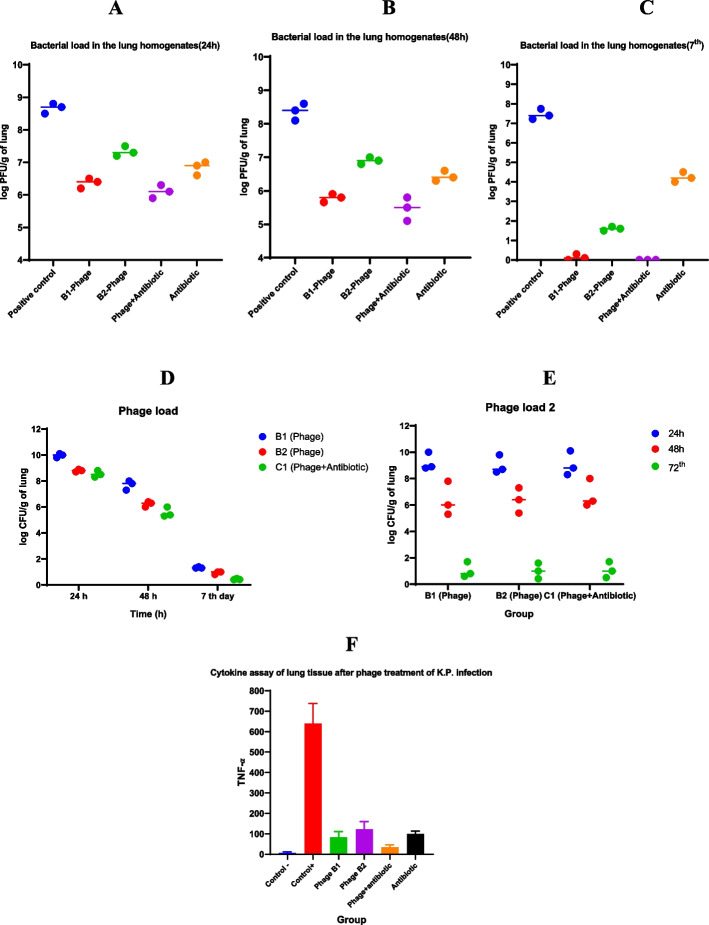
Average load of bacteria groups at 24 h (**A**), 48 (**B**), and 7^th^ (**C**) day post challenge, and average load of bacteriophage (**D** and **E**) in the lung homogenates of 3 mice per group. Bacteria load was significantly lower in the lung homogenate from the mice in the phage-treated group than in the control group. (*P* = 0.009 and *P* = 0.01). Bacteriophage load was significantly higher in the infected group than in the non-infected animals (*P* = 0.002). **F** TNF-α (pg/mL) levels of mice in different groups in 7^th^ day post challenge

Bacterial burden in the homogenate of lungs of group A mice showed significantly higher (6 × 10^8^ CFU/g) in comparison to other groups throughout the 24, 48 h, and 7^th^ day after the challenge (Fig. 6A-C). As well, the lowest amount of bacteria was isolated from the lungs of groups B1 and C1, respectively. The phage titer in the lungs of mice of B1 was higher than B2 and also both groups were more than group C1 (Fig. 6D, E). The spot test showed the highest amount of phage in the lungs in the first 24 h, and from the second 24 h began to decrease. Furthermore, phage titer in serum was demonstrated by spot test up to 24 h only in mice of groups B1(10^2^pfu), and after 24 h was not detected.

#### Cytokine assay of serum after phage treatment of BhvKp infection

Successful phage therapy also correlated with the production of TNFα (Fig. 6F). On 7th day post-challenge, TNF-α levels (pg/mL) in the phage-treated infected groups (B1 and B2) were significantly lower than in the un-treat group (A) and group C2, but still slightly higher than the synergism group (C1). Thus, the phage may be advantageous to the lung by subtractive the host inflammatory response, as represented by the levels of TNF-α.

### Hematological analysis

The hemocytometer counted sub-populations WBCs (Lymphocytes, Neutrophils, Monocytes) and Platelets (Table 2). Hematological analysis in group A, which was pneumonia model herein, revealed severe leukocytosis, neutrophilia, and thrombocytopenia. Groups B1, C1, and B2 that were treated with PSKP16 showed fewer blood neutrophils count compared to gentamicin treated mice. On 3rd day, total WBC count median was phage-treated mice without any significant difference among groups (*p* > 0.05).

**Table 2:** Evaluation of blood parameters 72 h after the challenge

Parameter	Hematological reference values	Result of normal BalB/c Mice	group A (× 10^6^ mm^3^) Median ± SD	Group B1 (× 10^6^ mm^3^) Median ± SD	Group B2 (× 10^6^ mm^3^) Median ± SD	Group C1 (× 10^6^ mm^3^) Median ± SD	Group C2 (× 10^6^ mm^3^) Median ± SD
Leukocytes (× 10^3^ /mm^3^)	1500–4800	2.9 (× 10^6^ mm^3^)	6.160 1.15	3.430 1.16	3.950 0.84	3.350 1.5	5.100 1.04
Segmented Neutrophils(/mm^3^)	300 – 900	0.55 (x10^6^mm^3^)	4.46 1.2	0.85 0.4	1.51 0.3	0.79 0.5	2.55 0.2
Monocytes (/mm^3^)	0 – 200	0.048 (x10^6^mm^3^)	0.031 0.01	0.044 0.02	0.043 0.01	0.04 0.01	0.040 0.02
Lymphocytes(/mm^3^)	900 – 3200	2.084 (x10^6^mm^3^)	1.370 1	2.309 1	1.870 0.8	2.2 0.85	1.976 1.15
Platelets (× 10^3^/mm^3^)	325–888	590	387 44.3	612 48	610 45	670 188	420 23
Red blood cells (× 10^6^/mm^3^)	7.1 – 9.5	8	7.93 1.1	8.19 1.05	8.05 1	7.59 1.1	7.81 0.9
Hemoglobin (g/dL)	11.6 – 15.8	12	12.1 1.3	13.3 1.7	13.1 1	12.6 1.2	11.9 0.6
MCV (fL)	41.5 – 57.4	51	46 5.56	53.8 7.6	46.8 6.02	52.9 4.7	49.9 6.02
MCH (pg)	14.1 – 18.4	16	15.4 1.35	16.1 1.75	15.9 1.27	15.9 2.1	16 1.3
MCHC (%)	30.5 – 34.2	32	27.3 3.7	32.2 1.5	32 1.3	31.6 1.7	31.8 0.72

*WBC* White blood cell, *RBC* Red blood cell, *MCV* Mean Corpuscular Volume, *MCH* Mean corpuscular hemoglobin, *MCHC* Mean Corpuscular Hemoglobin Concentration, platelet

The significant decreases in blood platelets and lymphocyte counts were only seen in the first 24 h' post-challenge but; in groupB1 were associated with mild changes (not statistically significant). The percentage of the more representative WBC sub-populations and Platelets was found to be similar both in B1 and C1-challenged mice, thus indicating that there is not any significant difference (*p* > 0.05) between the two treatment techniques. Moreover, the treatment with gentamicin (group C2) did not notably affect the percentages of these blood cells compared to PSKP16-administered mice, thus indicating the inflammatory response, induced by the infection, was not hampered by the antibiotic treatment. Moreover, the other routine blood parameters did not differ significantly between the treated groups and group A (*P* > 0.05) (Table 2).

Commonly used diagnostic biomarkers in clinical laboratories including C-reactive protein (CRP) and Procalcitonin (PCT) were not statistically different between groups of B1, B2, C1, and C2 on the 7th day and 48 h after infection, respectively. As for group A, the mean level of CRP and PCT were very high than other groups in all of the days of monitoring.

### Phage treatment improved the lung damage (Pathology)

**Fig. 7:**
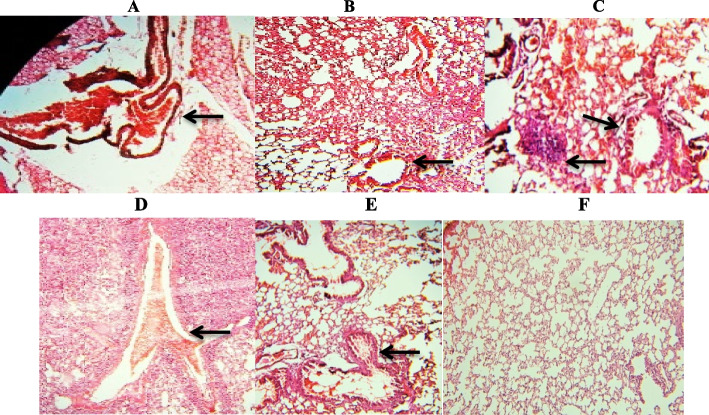
Sections of pathological changes micrograph of lung in challenge BALB/c mice groups (3 mice/group/timepoint), from group A (**A**–**E**) and group B1 (**F**) sacrificed at 48 h and 7^th^ day post challenge (haematoxylin and eosin stain). **A** Lung—Hemorrhage in mice of group A: **A** Erythrocytes are present in the perivascular space around a pulmonary vein at 48 h post challenge. **B** (48 h), **C** and **E** (7^th^ day), Necrosis of the alveolar parenchyma with interstitial infiltration of neutrophils, and macrophages. **D** Congested blood vessels with moderate to severe infiltration of neutrophils in the lung parenchyma. **F** Lung sections from Bhvkp-infected mice which was recovered by treatment with phage PSKP16 in 7^th^ day post challenge. Decreased white blood cells were observed

In histopathological analysis, leucocytes noticed in the lungs of mice were evaluated with the objective to assess the inflammatory cell recruitment in the lung. The high number of lung WBC infiltration in mice of group A Compared with inflammatory response level in all treatment groups along with PSKP16 was impressive. Survey of the presence WBCs sub-population in lung pathology indicated that the inflammatory response was mainly sustained by neutrophils and monocyte infiltration in the lung. Specifically, intra tracheal infection of group A with BhvKp showed wide bronchopneumonia, neutrophils infiltration, and suppurative with numerous infiltrating polymorphonuclear leukocytes (PMN) causing inflammation with alveolar edema and necrosis. At 7th day post challenge, in group A, the pulmonary alveoli showed an unusual structure, and the main part of the alveolar space was obliterated by inflammatory exudate and immune cell infiltrate, which was accompanied by hemorrhage consolidation. At 7th day, in the treated mice model by PKSP16, either individually or in combination with gentamicin, in terms of pathology revealed focal consolidation regions but in contrast, in the groupA mice model, diffuse consolidation was noticed. The local separate lesions were noticed in group B1 but most of the tissue was healthy and was remarkably less peripherally diffused inflammation in lungs. The spreading of inflammation to the environs along bronchi and large vessels was less pronounced in groups of B1 and C1 mice than in groups of B2 and C2 mice at 7^th^ day, as well as the severity and expansion of lesions were less obvious. The 7^th^ day was remarkably less eminent in lungs from PSKP16 phage-treated mice with less peripherally diffused inflammation (Fig. 7; Table 3).

**Table 3:** Comparing of grading of histopathological lesions in lungs and comparing therapeutic potential of phage with antibiotic-phage treatment. mild ( +), moderate (+ +), severe (+ + +) and widespread (+ +  + +)

	Time post infection	Group	Groups		Groups	
		A	B1	B2	C1	C2
Necrosis of alveolar	48hpi	+ + + +	+	+ + +	+ +	+ + +
	7^th^ dpi	+ + + +	-	+ +	+	+ +
Bronchiolitis with inflammatory exudate in lumen	48hpi	+ + +	-	+ +	+	+ +
	7^th^ dpi	+ +	-	+	-	+
Infiltration of inflammatory cells	48hpi	+ + + +	+ +	+ + +	+ +	+ + +
	7^th^ dpi	+ + +	+	+ +	+	+ +
Peribranchial and perivascular infiltration	48hpi	+ + +	+	+ + +	+ +	+ + +
	7^th^ dpi	+ + +	-	+ +	+	+ +

## Discussion

The crisis of the spread of antimicrobial resistance has been attributed to the misuse of these agents and the unavailability of newer drugs, which require novel methods to be seriously considered as potential therapeutic or preventive agents [19]. The World Health Organization (WHO) has declared a critical priority to develop new antimicrobials against carbapenem-resistant and ESBL-producing Enterobacteriaceae [20]. Phage therapy, as an effective and safe treatment, has been noticed again for patients who did not respond to antibiotics alone [21]. Nevertheless, a small number of phages have been described as specifically acting against *k. pneumoniae* strains with the virulent K2 serotype [22]. It is difficult to destroy BhvKp with regular medications type of bacteria, and it may become a superbug in the near future. Phage therapy is a replacement for antibiotics and studies notice that it is successful in circumventing bacterial resistance. However, an important concern is that bacteria can also develop resistance to phages [23]. In this study, resistance against the lytic activity of PSKP16 has been not reported in both in vitro studies and in vivo treatments, which is a desirable trait. Forasmuch as a new *K. pneumoniae* lytic phage (PSKP16) was isolated, that had a lytic effect on four MDR /XDR *K. pneumoniae* with K2 serotype; it is possible that the phage receptor is located on the K2 capsule. Similar to PSKP16, other phages that were from *Siphoviridae* family-specific for *K. pneumoniae,* display an isomeric head of 45–80 nm diameter and a long non-contractile tail of 90–190 nm long [24]. PSKP16 has specific features, that potentially can be used as a bacterial treatment tool, confirmed by its high antibacterial activity.

Enzymes such as depolymerase, endolysins, and virion-associated lysine in some phages can cause the degradation of biofilm, as a result becomes much easier to eradicate by antibiotics and the host immune system [25]. In some studies direct effect of phage on biofilm structure has been demonstrated in *Klebsiella* [26], and *P. aeruginosa* [27]. Herein, a characteristic biofilm degradation potential was observed for PSKP16 using the standard method of phage-bacteria co-cultures set up at different MOIs, in the CV assays. Similar to our study in some studies the desirable anti-biofilm activity of phage was indicated, which was dose-dependent [28]. Within this study, we noted a marked increase in phage susceptibility of the BhvKp biofilm to PSKP16 with increasing age but, there was no significant change after 24 h to 48 h (*P* value < 0.05). Nevertheless, the presence of phage prevented the growth of the biofilm. A study proved, there is a significant change in the density of the treated biofilm with a lytic phage alone, after 4–5 days [26]. In contrast, other studies of phages have demonstrated that a higher concentration of phage treatment does not always result in a greater magnitude of biofilm removal [29]. Assumptions including phage different bactericidal activity, lack of phage stability, and biofilm disruption as occurs in vitro thus may be questioned should seemingly adequate phage dosing but resulting in treatment failure [29, 30]. Generally, further understanding of phage-biofilm interaction at molecular level may prevail the clinical challenges in phage therapy [31]. The optimal pH and temperature for phage PSKP16 were 7 and 25 °C, respectively. The durability of phage PKSP16 under different temperature and PH conditions showed similarity with other published members of the *Siphoviridae* families [32]. The reduction of bacterial load in infected hosts due to the bactericidal effect of phage is a known phenomenon [33]. Herein, the number of live bacteria and phage in the lungs, and pathological changes in the lung, in experimental models corresponded to this phenomenon. Nevertheless, not all phage therapy cases have culminated in positive clinical outcomes [34, 35] and exactly which functional characteristics or specific structural of a phage enhances its therapeutic efficacy, is left undefined [36]. Analogously, short time to lysis induced by phages may be a predictor of phage-appropriate efficacy in cases of Gram-negative bacteremia [36]. In our study, the shorter latent period of PSKP16 may account partly for the enhanced survival of PSKP16-treated mice. Our finding demonstrated that the prompt and early administration of phage rescued more infected mice than late administration at 24 h post-challenge. It could be argued that, prophylactic bacteriophage administration is more effective than after the infection has developed, in reducing BhvKp strain. Considering that these capsular serotypes (K1 and K2) rapidly become systemic infections [22], therefore, the speed of infection control using phage therapy methods with PSKP16 is beneficial. It was also shown that after 24 h from the challenge, a combination therapy of phages and antibiotics is better than one alone.

The efficacy of prompt PSKP16 administration (group B1) to decrease bacterial load and survival, was faster than late synergism administration (group C1), but the two displayed almost similar endpoints. Several bacteriophages have been introduced to treat pneumonia that successfully controlled bacterial pulmonary infection by inhalation with phages [37, 38]. Consistent with other studies [36, 39], our result showed that the nasal inhalation of concentrated PKSP16 (MOI = 10) significantly enhanced the survival rate in pneumonia models (*P* < 0.01). Diagnosis of bacterial pneumonia relies on, recognition of lung septic inflammation in conjunction with a positive bacterial culture [18], which was confirmed in this study. Mice were monitored closely for pneumonia clinical symptoms, and vital signs, body temperature, weight, CBC, and lung bacterial burden were surveyed for 7 days. Herein, we applied two treat-assay, by using mice for the immediate and delayed treatments; and the effect of the phage strongly was dependent on the time between bacterial inoculation and phage administration. Specifically, the on-time administration of phage led to perfect protection from mice of group B1 but late administration led to delayed death in group B2, only. Based on the fact that the BhvKp strain has a K2 capsule, and is indispensable for in vivo survival of *K. pneumonia* [40], immediate phage therapy showed that it could limit *K. pneumonia* K2 serotype proliferation in the lungs, and significantly decrease mortality. In determined follow-ups post-challenge, considering that in the first days, PSKP16 was associated with higher titer in the lungs of the mice B1 group compared to groups B2 and C1, it was noticeable that survival was also, higher among group B1. It is speculated that phage inoculation 24 h after bacterial colonization and the creation of opportunity for its proliferation in vivo, results in poor phage therapy. The MOI is an important parameter when using viral expression systems. Usually, a lower MOI reduces the probability of both transducing and infectious phage particles entering the same recipient cell which may reduce the numeral of transductants killed [41]. In this case, it is conceivable that the phage has been eliminated before it is attached to the host and cracked it. As well as, the clearance rate of the phages from body fluids by the reticuloendothelial system is a main parameter for phage therapy [41]. Although shown, a small dose of phage can achieve an excellent therapeutic effect as phage proliferation via auto “dosing” results in great bacterial killing [42].

We present evidence of lower viable bacterial burdens in the lung of mice of groups B1, B2, and C1, which were significantly lower than that of group A at 7th dpi, respectively. Herein, it seems that, in the sizeable bacterial population, phages can increase to a greater extent than the condition of adding phages along with gentamicin. This is our inference, although phage and antibiotics can act in synergy to kill bacteria, reducing bacterial growth by antibiotic, may also, reduce phage production. In this regard, a study stated that, the phenomenon of phage–antibiotic synergy, in which antibiotics may induce the decrease of production of phages by bacterial hosts has been observed [43]. Phage therapy along with antibiotics, should is investigated well, in varying timings and concentrations of phage and antibiotics. Owing to maybe led to a slower clearing rate of bacteria and or a higher risk of MDR evolution [43]. Another study indicated that a critical determinant of the remedy of mice is the on-time administration of phage accompanied by doses of effective antibiotics [20]. Although some reports did not have favorable results, which can be due to phage selection having been suboptimal in those cases, or if circumstances were unfavorable for other reasons [35, 44]. As a result, phage PSKP16 cannot alone provide significant protection after 24 h inoculation of bacteria. This treatment method may be more effective with increase the dose of phage or giving phage less than 24 h after inoculating bacteria. However, our models were not designed to fully answer this aspect of the questions. The high pathogenicity of BhvKp pathogen necessitated a phage administration simultaneous to get-infection to preserve a high survival rate of treated animals, similar to other studies [22, 45]. Regardless of clinical surveys, all mice groups except group D, lost body weight between 5 and 15%, similar to other studies [22, 46]. Our observation indicated PSKP16-gentamicin synergism was more effective than late phage or antibiotic administration, alone, even if performed 24 h after inoculation of BhvKp strain. Clinical status characterization, alleviation of symptoms, and bacterial lung burden in groups B1 and C1 were almost comparable, but was happening slower in group C1. Nevertheless, synergism and Gentamicin-treated mice, due to antibiotic use, showed higher body weight loss compared with phage treated-mice. In general, all phage therapy cases have culminated in positive clinical ends in this study. Such that, here, phage therapy was not associated with over-inflammation, in contrast, tended to lower inflammation and provided a faster correction of blood cell count abnormalities than did C1 and C2. The CBC is a useful diagnostic test in animals with respiratory symptoms. Bacterial pneumonias are often associated with an inflammatory leukogram, characterized primarily by a neutrophilia [47]. Neutrophils is as the prime infiltrating and short-lived cells that is necessary for combating pneumo-septic infections including *K. pneumonia* [48, 49]. Although in the first days after infection, except in group A, the level of inflammation and the number of neutrophils was also noticed in other groups. However, during the treatment of mice by PSKP16, inflammatory parameters were strongly reduced, whereas, in gentamicin-treated mice, just was noticed a limited reduction in inflammation, despite bacterial load control. In one study believed phages may also be capable of modulating the immune response in a phage-specific manner [50]. Groups B1, C1, and B2 that were treated with PSKP16 had a more decrease in neutrophils compared to gentamicin-treated mice. The findings suggest that phages may cooperate synergistically with phagocytes in killing bacteria and modification of the immune response. Treatments that reduce neutrophils number like phage therapy may be of benefit [51].

Generally, hematological changes in the phage-treated groups were mild or less severe, and changes were shorter duration than in groups A and even C2. According to the clinical symptoms and lung pathological section results, group B1 and then group C1 had significantly lower damage, and a small amount of collapse in local alveolar walls, but most of the alveolar structures were yet at their norm. Other studies also, showed the low histopathological changes in the phage-treatment groups [52, 53].

As an important pro-inflammatory cytokine, TNF-α is a key factor in the pathophysiology of cytokine release syndrome (CRS), also known as a cytokine storm [54]. Successful phage therapy also correlate with the production of TNFα [54]. On the 7th-day post-challenge, TNF-α levels in the phage-treated infected groups were significantly lower than in the un-treat group but still high than in the synergism group. The involvement of the immune response in the success of respiratory phage therapy was previously demonstrated and modeled [55]. Another study showed phages targeting host strains regulate pro-inflammatory and anti-inflammatory cytokines in mice [56]. Our data indicate an apparent presence of pathogenicity of hypervirulent *K. pneumoniae* isolate of the capsular serotype K2, which is among the most virulent and potentially considered as pathogenic. It can be interpreted that the mice's immune response being nonsufficient to clear this highly virulent strain. PSKP16 reduced BhvKp in the lungs regardless of the pathogen and reduced inflammation of mice infected with strain BhvKp. We cannot strongly reject this hypothesis that, phage acted faster than antibiotic because indirect data (lung weight, lung appearance, pathology results, and bacterial load reduction), and inflammatory marker levels of infected—mice exposed to phage PSKP16 will support it.

## Conclusion

This study suggests that a phage treatment that is administered intranasally has great potential for treating pneumonia and probably other infections caused by the superbugs like carbapenemase-producing/ESBL hvKp strains. These strains with high virulence factors can become a serious challenge. phages with a narrow host range are not harming the microbiota but antibiotics not only kill the target bacteria but might also disrupt the host's normal flora and ecological balance. In addition, herein, phage therapy did not affect the weight of mice but was seen a significant decrease in the weight of the group mice that received antibiotic. PSKP16 prompt administration caused phage to proliferate rapidly, rescued all the infected mice, and completely eliminated the bacteria in the lungs within 7 days, which exceeded the therapeutic effect of gentamicin alone. Also, the late administration of the combination of PSKP16 and gentamicin rescued more infected mice than if their administration alone.

## Materials and methods

### Bacterial isolation and identification

From October 2021 to March 2022, 30 non-duplicate *K. pneumoniae* respiratory isolates were collected from 123 bronchoalveolar lavage (BAL) samples of suspected cases of ventilator-associated pneumonia (VAP) that were adult patients hospitalized in intensive care units (ICU). These 30 K*. pneumoniae* (27 patients, 19(70.3%) male, 57 ± 9 years),) analyzed as per standard protocols and was used as host bacteria to determine the host ranges of phages. From MacConkey agar, mucoid colonies and red pigment were picked up and subcultured onto TSA at 37 °C for 24 h, then performed biochemical identification using API 20 E (bioMérieux, France) strips. Afterward, isolates were confirmed to be *K. pneumoniae* using 16S rRNA sequence analysis after PCR amplification with the universal primers (27F: AGAGTTTGATCCTGGCTCAG and 1492R: GGTTACCTTGTTACGACTT) [57]. Isolates were stored at − 20 °C in TSB containing 20% glycerol until further studies.

### Phenotypic analyses

#### Antimicrobial susceptibility testing

Antimicrobial susceptibility test was performed by the disk diffusion methods according to the Clinical and Laboratory Standards Institute (CLSI; 2022) [58]. Antibiotic disks include levofloxacin (LEV) (5 µg), azithromycin (AZ) (15 µg), ceftazidime (CAZ) (30 µg), amikacin (AN) (30 µg), gentamicin (GN) (10 µg), cefepime (FEP) (30 µg), imipenem (IMP) (5 µg), meropenem (MEN) (5 µg), piperacillin-tazobactam (PTZ) (100/10 µg), ciprofloxacin (CP) (5 µg), cotrimoxazole (SXT) (25 µg), cefotaxime (CTX) (30 µg), and cefoxitin (FOX) (10 µg) [59]. The MICs of colistin and tigecycline were determined using the broth microdilution method, and the results were interpreted based on the European Committee on Antimicrobial Susceptibility Testing (EUCAST) breakpoint recommendations [60]. *Escherichia coli* ATCC 25922, *K. pneumoniae* ATCC 700603 were included as negative and positive controls for ESBL production, respectively [61]. Moreover, MDR isolates were defined as no susceptibility to at least one agent in three or more antimicrobial categories, and XDR was defined as non-susceptibility to at least one agent in ≥ 6 antimicrobial categories [62]. The ESBL/AmpC phenotypes were interpreted according to CLSI 2022 breakpoints [59].

#### Biofilm formation assays

The bacterial isolates were inoculated with turbidity equal to 0.5 McFarland (1.5 × 10^8^ CFU mL − 1). A 200-µL suspension was incubated in each well at 35 °C. After 24 h, the wells were washed three times with phosphate buffer. Following incubation with 1% crystal violet dye (200 µL/well) at 25˚C for 20 min, the wells were washed three times with phosphate buffer and dried. Finally, Ethanol 95% (200µL/well) was added, and optical absorbance was measured at 550 nm (Thermo Scientific GmbH, Driesch, Germany) [63]. *P. aeruginosa* ATCC 27,853 was used as a positive control, and LB medium was used as a negative control. Biofilm production was classified as: OD < ODc = negative biofilm; ODc < OD ≤ 2 × ODc = weak biofilm producer; 2 × ODc < OD < 4 × ODc = moderate biofilm producer; and OD ≥ 4 × ODc = strong biofilm producer [64]. OD: Optical Density, ODc: Optical Density control.

#### Hyper virulent K. pneumoniae phenotypic identification (String test)

BhvKp isolates were incubated overnight on blood agar at 37 °C. A single colony was touched with a loop and stretched outward. The hypermucoviscous phenotype of the HvKp isolates was tested by the string test. The string test was deemed positive when the formation of > 5 mm viscose filament, by using an inoculating loop to stretch a colony grown on a blood agar plate [65].

### Genomic analysis

#### DNA extraction and detection of carbapenemases, ESBL, and AmpC genes

Briefly, bacteria were grown overnight in Mueller–Hinton broth (Merck, Darmstadt, Germany) at 37 °C and genomic DNA was extracted using a high Pure PCR template Prep kit for Genomic DNA extraction (Roche Diagnostics, Mannheim, Germany) according to manufacture instructions. The β-lactamase genes (*bla*_TEM_*, bla*_SHV_, and *bla*_CTX-M_), carbapenemase genes (*bla*_IMP_*, bla*_VIM_*, bla*_NDM1_*, bla*_KPC_, and *bla*_OXA-48-like_) and AmpC cephalosporinases genes (*bla*_ACC_*, bla*_DHA_*, bla*_EBC_*, bla*_FOX_*, bla*_MOX_*,* and *bla*_CIT_) were determined by PCR as previously described (primers showed in Table S2).

#### Detection of virulence-associated genes and capsular serotype-specific (cps) Genes for β-lactamase-producing K. pneumoniae strains

In this study, HvKp could be defined as: positive capsular types K1, K2, K5, K20, K54, and K57, positive siderophore genes ≥ 2 (*entB, iutA, iroB, ybt*S and *iucA*), or ≥ 1 positive capsule-regulating genes (*prmpA, magA, wcaG*) and positive adhesion (*mrkD, fimH*). Non-hvKp is termed as CKp (classic *K. pneumonia*) [66, 67].

Genes of capsular serotypes K1, K2, K5, K20, K54, and K57 and virulence-associated genes *rmpA, fimH, mrkD, iutA, iucA, magA, wcaG, entB, ybt*S and*, iroB* were screened by PCR method (The primers used are summarized in Table S3). The amplified transcripts were sequenced, and BLAST was used to determine their identities.

#### Determination of multilocus sequence typing (MLST)

MLST was used to investigate the sequence types (STs) of the total BhvKp isolates. Briefly, the housekeeping genes *gapA, infB, mdh, pgi, phoE, rpoB*, and *tonB* were amplified by PCR and sequenced [68]. The STs were identified by the MLST database (https://bigsdb.pasteur.fr/klebsiella/klebsiella.html). All primer sequences used in MLST are listed in Table S4. In addition, capsular types were determined for BhvKp isolates.

#### Bacteriophages isolation, purification and determine host spectrum

Altogether twelve samples from hospital sewages were collected and stored at 4 °C for 24 h to precipitate their suspended solids. The waste, debris, and soil in the sewage were eliminated by centrifugation at 10,000 × g for 15 min, and then the supernatant was passed through a 0.22 μm membrane filter (Millipore, USA) to eliminate bacteria. For bacteriophage enrichment, 10 mL of samples was mixed with 50 ml Luria–Bertani broth (LB) and added *K. pneumoniae* isolates (1.5 × 10^8^ CFU/ml) with further incubation at 37 °C with shaking. Following incubation, the enriched broth was centrifuged at 10,000 g for 10 min and was filtered through a 0.22 μm membrane filter [69]. Bacteriophage activity was detected using the spot assay. Phage titer in the filtered sewage samples was about 10^3^ PFU per ml for PSKP16.

Phage PSKP16 was selected and purified by the double-layer agar plate method and then stored at 4 °C or − 80 °C in glycerol (3:1 *v/v*). Single plaques were identified from the plaque assay plates, picked using a pipette tip, mixed with 50 µL of nutrient broth, and filtered through a 0.22 µm spin filter. The filtrate underwent two further rounds of plaque assay to certify that plaque isolates resulted from a single clonal phage [70].

To determine its host spectrum, 5 μL of phage PSKP16 suspension was spot tested against Bhvkp strains preserved in the laboratory, by the double-layer agar (DLA) plate method. The DLA method was used to confirm the presence of lytic phage in the supernatant. One clearly separated plaque was purified three times by picking in SM buffer (5.8 g/L NaCl, 2.0 g/L MgSO4, 50 mL/L of 1 M Tris, PH 7.5, 5 mL/L pre-sterilized 2% gelatin) and re-plating. Single plaques were picked using sterile Kimble glass Pasteur pipets, inoculated into 5 ml of LB broth, or followed by the addition of 10 μl of HvKp (1.5 × 10^8^ CFU/ml). The tubes were incubated for 24 h followed by centrifugation of the cultures (6000 rpm, 10 min) and were then filtered (0.22 μm filters) [71]. The harvested phages were selected according to their plaque morphology and were picked and suspended in LB broth, separately. Then, phages were stored in the refrigerator at 4 °C for further analysis. To determine the host range [72] of the phage isolated in this study, strains belonging to the species of *Escherichia coli* ATCC 25922, *Pseudomonas aeruginosa* ATCC 27853, ESBL producing *K. pneumoniae* ATCC 700603, *Enterococcus faecalis* ATCC 29212, *Acinetobacter baumannii* ATCC 24637, *Staphylococcus aureus* subsp. aureus ATCC 25923, *Shigella* ATCC 12022, and *Salmonella enterica* subsp. enterica serovar Typhimurium ATCC 14028™ were used for spot test. The host range of PSKP16 phage was determined by spot testing on 30 K*. pneumoniae* isolates possessing known capsule serotypes (Table S3). For the spot test, an aliquot of 500 µl of freshly cultured bacteria was added to 3 ml of 0.7% top agar and poured on a 1.5% LB agar plate. After solidification of the top agar, 5 µl of bacteriophage suspension was spotted on the plates and incubated at 37 °C, overnight. The presence of a clear zone and lysis plaque was recorded as the strain is susceptible to the tested phage. The host range was determined in a single experiment with technical triplicates.

### Electron Microscopy observation of phage virions

Identification and classification were performance according to the size, tail structure, and head structure of the phages by Transmission Electron Microscopy (TEM). Therefore, the phage plaques formed on the agar plate were washed off by using an SM buffer (10 mM Tris, 100 mM NaCl, and 10 mM MgSO4, pH 7.5). The phage was negatively stained by 2% uranyl acetate on a mesh copper grid with carbon-coated formvar film and visualized under Zeiss-Em10c-80 TEM [73].

### Thermal and PH stability

In order to test the phage stability ranges, we exposed the phage to diverse PH and temperature positions. To determine the PH stability, 2 × 10^8^ PFU/ml phages were mixed in sodium acetate buffers (pH 3 to 6) and 1 M Sodium hydroxide (NaOH) (PH 7 to 11) in the ratio of 1:10 (v/v) and incubated at 37 °C for 1 h. Then, the phage titer was tested using the DLA method [74]. For the temperature stability test, bacteriophage suspensions of (1 × 10^8^ PFU/mL) were incubated at 4 °C, 20 °C, 25 °C, 37 °C, 50 °C, 60 °C, 70 °C, and 80 °C (PH 7) for 1 h, and then, the phage titer was tested using the DLA method. For assessment of on-shelf activity over a longer duration, the phage suspension was placed at 37 °C and was assessed for activity by spot test after every 10 days [53]. The above experiments were performed in triplicate [53].

### Sequencing and bioinformatics analysis of the phage genome

The phage DNA was extracted from 1.5 mL purified phage suspensions with titers of 10^9^ PFU/mL, using a viral genome extraction kit (NorgenBiotek, Thorold, ON, Canada). Whole-genome was sequenced by using Illumina technology by MicrobesNG (Birmingham, United Kingdom). Here we compare the sequence quality obtained using libraries prepared from the Nextera XT and Nextera DNA Prep (Illumina, San Diego, CA). Input DNA is increased twofold, and PCR elongation time is increased to 45 s. DNA quantification and library preparation are carried out on a Hamilton Microlab STAR automated liquid handling system (Hamilton Bonaduz AG, Switzerland). Pooled libraries are quantified using the KapaBiosystems Library Quantification Kit for Illumina. Libraries are sequenced using Illumina sequencers (HiSeq/NovaSeq) using a 250 bp paired-end protocol [75, 76]. Raw reads were adapter trimmed using Trimmomatic 0.30 with a sliding window quality cutoff of Q15 [77]. The reads were de novo assembled using SPAdes version 3.10.1 [78]. We standardly used Prokka, version 1.14.6, for the annotation of phage genomes, using the PHROGS database [79].

Open reading frames search was performed using ORF finder National Center for Biotechnology Information (NCBI). BLASTN (https://blast.ncbi.nlm.nih.gov/Blast.cgi) was used to align genes of PSKP16 with other bacteriophages, calculate nucleotide identity, and query coverage. The Schematic map of phage Pspk16 genome prepared using CGView. Taxonomic identification was made by GenBank (https://www.ncbi.nlm.nih.gov/genbank/) based on the phylogenetic classification scheme used in the NCBI Taxonomy Database (https://www.ncbi.nlm.nih.gov/Taxonomy). Phylogenetic trees of phages were constructed using Neighbor-Joining Method (100 bootstrap replicates) by Mega (version 11). The complete genome sequence and associated data for K. *pneumoniae* phage PSKP16 were deposited under GenBank accession number OW251746.1, BioProject accession number PRJNA833576, SRA accession number SRR8869225, and BioSample accession number SAMN27615979.

### Phylogenetic analysis of phage PSKP16

Whole genome-based phylogenetic analysis was conducted with MEGA11 involving the first 40 highly similar *Klebsiella* phages, according to the homology searches and BLAST in NCBI. All pairwise comparisons of the nucleotide sequence were conducted using the genome–BLAST distance phylogeny (GBDP) method, under settings recommended for prokaryotic viruses. Branch support was inferred from 100 pseudo-bootstrap replicates each.

### Biofilm disruption assay by phage

Two types of phage treatments with phage concentration of 10^1^–10^8^ PFU/well were performed to check the susceptibility of biofilm against bacteriophage PSKP16 by Crystal violet Assay (CV assay): Post-treatment of phage infection and pre-treatment of phage infection was performed using a previously published protocol with slight modification [80]. The concentration of BhvKp strain was determined to be 1.5 × 10^8^ CFU mL − 1 for biofilm formation.

### Colony forming unit (CFU) assay to quantify viable cells in pre-formed biofilm

Before the pre-formed (mature) biofilm disruption experiment by PSKP16, to total count of live bacterial cells in the case of pre-formed biofilms and set up MIO to remove the pre-formed biofilm, was used the Colony-Forming Unit (CFU) spot test. The wells with biofilm were cut, transferred to 200 µL of sterile saline, and sonicated for 30 min. Subsequently, the bacterial suspension was serially diluted, and 10 µL of each concentration was placed on TSA plates. After 16–20 h of incubation at 37 ◦C, the colonies were counted, and the CFU values for viable cells in the pre-formed biofilm were calculated [81].

### Growth characteristics of the phage

We determine the optimal MOI by adding a certain amount of phage to a fixed number of cells. The BhvKp strains were grown in Nutrient Broth (NB) to mid-log phase 2 × 10^8^ colony-forming unit(CFU)/mL (OD600 ≈ 0.6) at different MOIs (phage/bacteria = 0.1, 1, 10). Bacterial growth was monitored by recording OD600 at 30 min intervals up to 15 h. After incubation for 3 h at 37 °C, the phage titers were measured by the DLA method to determine the highest number of phages as the optimal MOI [82]. A one-step growth test was performed by modifying the method described by Ellis and Delbruck [83].

### Bactericidal Effect of phage PSKP16 in Vitro and determination of optimal MOI

An aliquot of an overnight culture of BhvKp was cultured into fresh LB medium and incubated for 6 h at 37^∘^C with shaking (150 rpm). BhvKp (2 × 10^8^ CFU/mL − 1) was infected with the phage at different multiplicities of infection (MOI: 0.1, 1, 10). Phage PSKP16 action on bacterial (BhvKp) growth was observed through a change in OD600 over 12 h of incubation. Bacterial growth was monitored by measuring the OD600 at a 30 min intervals. Data from experiments set up with different MOIs indicated inhibition of bacterial growth due to phage action [83].

### Characterization of Bhvkp strain for use in a mice pneumonia model

This study described a mice model of pneumonia of a clinical isolate of beta lactamase-producing strain *K. pneumoniae* (hereafter referred to as Bhvkp) that was obtained from the BAL sample. The Bhvkp strain has the hypermucoviscous phenotype (string ≥ 5 mm) on the blood agar plate. This strain was belonging to the ST273-K2 lineage and was approved for attendance of a series of β-lactamase and virulence genes (Table 1). This strain showed that was resistant to all antibiotics tested, including imipenem, meropenem, and colistin, and was sensitive to gentamicin and tigecycline. Bacteriophage PSKP16 infected Bhvkp strain in vitro experiments. Then, the models induced by Bhvkp strains administered intranasally, have been analyzed not only in terms of infection development in the lungs of animals untreated and treated with gentamicin or phage PSKP16 or both gentamicin and PSKP16 but also has been analyzed by examining the lung inflammation through histopathological analysis and cytokines and leukocytes cells infiltration determination. Stocks of Bhvkp strain were stored at − 80∘C in LB containing 25% glycerol.

### Induction of pneumonia and therapeutic effects of PSKP16 in a mice model

#### Ethical clearance

All experimental protocols were approved by the Ethics Committee of Qazvin Medical University (IR.QUMS.REC.1398.410). The research protocol was approved by the Research Ethics Committee at the Qazvin Medical University, and by the committee on animal experimentation of the Institute Pasteur Iran. All experiments were performed with the criteria described in “Guidelines for Ethical Conduct in the Care and Use of Non-Human Animals” [84]. Based on the Declaration of Helsinki the welfare of animals used for research was respected. All methods were carried out in accordance with relevant guidelines and regulations. All methods are reported in accordance with ARRIVE guidelines (https://arriveguidelines.org). Phages were passed through a Detoxi-Gel endotoxin removing gel (Pierce Biotechnology, Rockford, IL) to remove endotoxins from the phage preparation.

#### Mice pneumonia model

BALB/c mice of the male sex (6–8 weeks old, weight 18–24 g) were purchased from the Razi Vaccine and Serum Research Institute of Iran and then weighed. Mice were acclimatized for at least 3 days before the start of an experiment. Food and drink were provided ad libitum, and cages were sterilized and replaced twice weekly. All mice were housed at the animal experimental center of Qazvin medical the university under pathogen-free conditions in a temperature-controlled room (23 °C). To enhance the accuracy of tests, the cages were disinfected with 10% povidone iodine (PI) solution, and the bedding materials for mice were autoclaved.

**Fig. 8:**
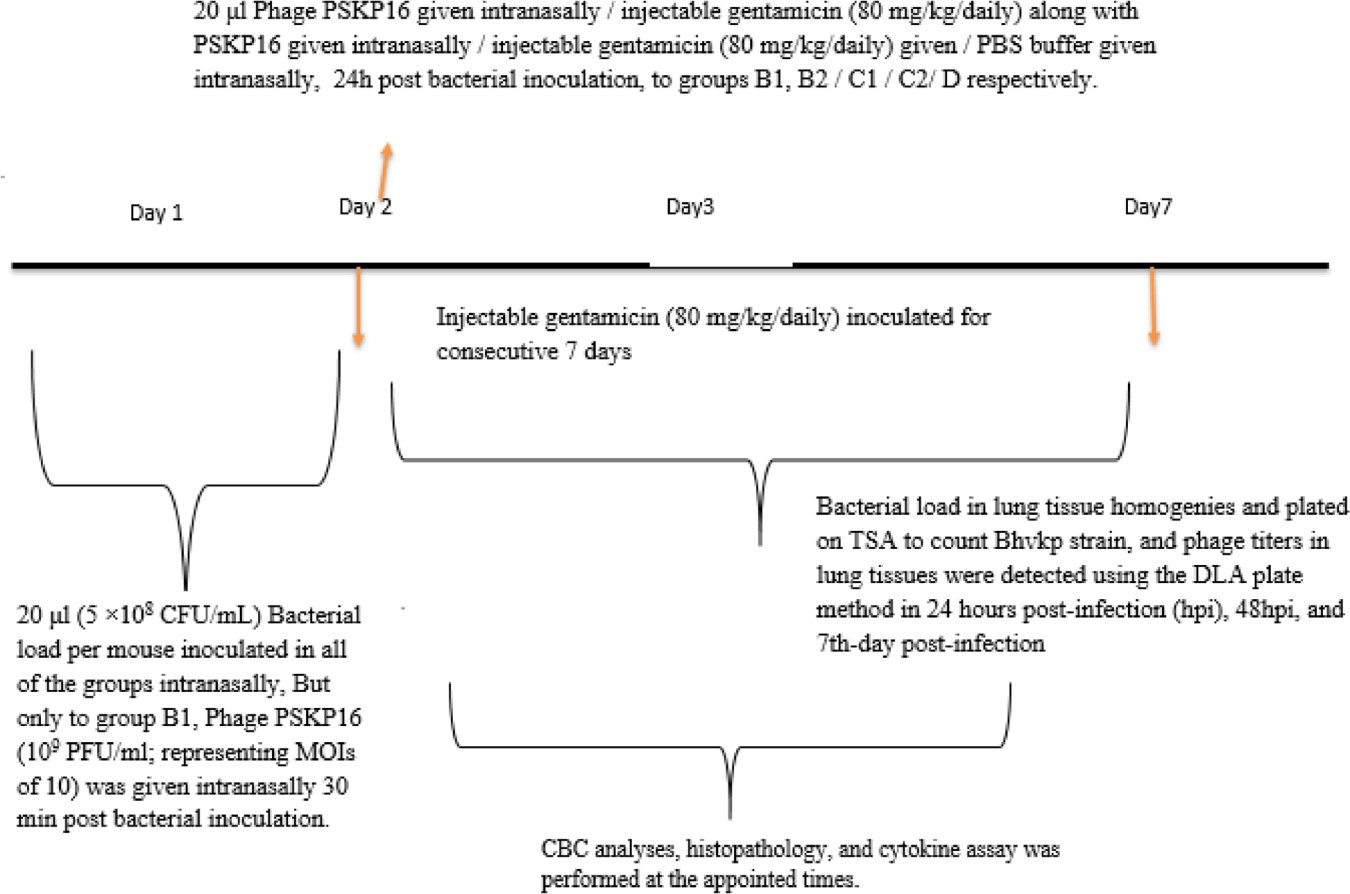
Graphical depiction representation of the infection and treatment schedule followed for setting up the intranasal colonization model in BALB/c mice

On the first day of the study, BhvKp isolate was propagated in TSB at 35 °C for 8 h (exponentially growing cells), then washed two times in sterile saline to remove the culture medium and re-suspended in sterile saline. The optimum dose for inducing pneumonia was established with 20 μl bacteria (5 × 10^8^ CFU/mL) of inoculum per mouse, which led to achieving persistent infection and animal lesions with no mortality. In groups intervened with bacteriophage, 20 μl Phage (10^9^ PFU /ml; representing MOIs of 10) was inoculated by the intranasal route performed for each experiment, at the appointed times [85]. We also report trends in survival differences between mice receiving 3 different types of phage treatments (time-dependent phage administration, combination phage and antibiotic administration and antibiotic therapy). We weighed mice and were divided into 6 major groups as follows (randomly allocated into every experimental group, n:12 per group): The descriptions of the groups are in the Fig. 8. These time points were chosen to match the phage used during experiments and totally with the gentamicin injection schedule to limit mice handling. The groups of mice were anesthetized by an IP (intraperitoneal) injection with ketamine (120 mg kg − 1) and Xylazine (12 mg kg − 1) [86]. On the scheduled days, the mice were then sacrificed using 25 and 250 mg/kg of Xylazine and ketamine, respectively.

On each specific day, the mice's weight was measured and the results were evaluated statistically. All mice were assessed concerning their health status such as; survival rate, sudden death, disease symptoms, body weight loss, depression, and decreased activity, then categorized by surface temperature into two groups (moderate (≥ 32 °C) and severe between 30 and 32 °C) within 16 day follow-up period. In these groups, the appearance of changes in the mouse hair was assessed. After pneumonia was induced, the surface temperature of the abdominal region was measured by monitored as before developing pneumonia a thermometer. Each trial was performed twice [87].

### Bacterial burden, complete blood Count (CBC) analyses, histopathology, and cytokine assay of lung tissue

To follow up on the efficiency of the phage in the treatment of pneumonia, the bacterial burden in whole-lung homogenates was counted [88]. Simultaneously, the phage titers in lung tissues were detected using the DLA plate method. This survey was conducted in groups B1, B2, and C1 on the 24 h post infection (hpi), 48hpi, and 7^th^ day post infection (dpi), and the results were evaluated.

In the part of blood analysis, for determination of the peripheral blood cell population following morphometric standards, blood smears were made every day post-challenge. The blood was collected from the tail vein of mice (about two-thirds down the tail, from the tail root), then blood smears on a glass slide were prepared and stained with Giemsa reagents [89]. Ten high-power (100 × oil objective) fields in the monolayer of the blood smear were examined for morphologic and quantitative changes in peripheral blood (Both red and white blood cell numbers (RBC and WBC)). The various leukocyte populations were recorded, also. The feathered edge was scanned at 10 × and 40 × for platelet clumps; platelet clumps within the monolayer were noted. The CBC analysis was performed using fresh EDTA-anticoagulated whole blood from all of the groups of mice, with a Hemavet veterinary hematology analyzer (that provides accurate CBC counts from the whole blood of mice, rats, and dogs). For this purpose, three mice were selected randomly from each group in 24 h (n:3) and 72 h (n:3) post-challenge. We compared these results to those obtained in control mice (n:3) that received PBS.

By a minimally invasive, low-stress method for blood collection, whole blood samples were aseptically collected via heart puncture, preferably the ventricle, through the diaphragm, or from the top of the sternum [90]. At the end, confirmed by peripheral blood smear. The sample volume obtainable was almost, 0.5 to 1.0 ml (3–5% of body weight). Whole blood was used for the determination of hemoglobin, count of WBC, RBC, platelet, and other of blood cell. Internal quality control was performed before analysis. These results from the original hematology analyses were reviewed for the present study.

At the predetermined time point for pathological study (48 h and 7th days after infection), after weighing, mice lungs were carefully removed and after fixation with 4% formalin, the lungs were embedded with paraffin and stained with hematoxylin and eosin (H&E) [28]. followed by histopathological analysis through a microscope. Histological parameters selected for analysis of the lesions included the extent of necrosis of the alveolar parenchyma and infiltration of neutrophils and lymphocytes. The scoring of the lung lesions was performed for quantifying and evaluating the variations between the lesions in infected mice vs bacteriophage-treated or gentamicin-treated groups on the basis of the most characteristic specifications observed in the lungs. Once collected, blood was centrifuged at 1500 g for 10 min at 4 °C to pellet blood cells and serum was subsequently frozen at -20 °C. TNF-α concentrations in the supernatants of serum were measured by ELISA kits (PeproTech, London, UK) according to the manufacturer’s instructions in 7^th^ day post challenge. In general comparison between the six groups was made based on observation of clinical, microbiological, CBC analyses, immunological, and histopathological examinations.

### Statistical analysis

Descriptive statistics were used to measure the characteristics of the study. If the distribution was normal or non-normal results were reported as mean ± s.d. or median (interquartile range (IQR)), respectively. Pearson chi-square, T-test, One and Two-way-ANOVA, and Liner regression test was used to determine significant differences between proportion. *P* values of < 0.05 were considered significant. Statistical analysis was performed by using Graph Pad Prism version 8.0.2 statistical software.

### Supplementary Information

**Additional file 1.**

## Data Availability

The complete genome sequence and associated data for K. *pneumoniae* phage PSKP16 were deposited under GenBank accession number OW251746.1, BioProject accession number PRJNA833576, SRA accession number SRR8869225, and BioSample accession number SAMN27615979. The datasets used and analyzed during the current study are available from the corresponding author on reasonable request.

## Abbreviations

BLAST: Basic Local Alignment Search Tool
CFU: Colony Formation Unit
CLSI: Clinical and Laboratory Standards Institute
CR-hvKP: Carbapenem-resistant hypervirulent *Klebsiella pneumoniae*
EUCAST: European Committee on Antimicrobial Susceptibility Testing
HvKP: Hypervirulent *Klebsiella pneumoniae*
*K. pneumoniae*: *Klebsiella pneumoniae*
*KPC*: *Klebsiella pneumoniae* Carbapenemase
MICs: Minimal Inhibitory Concentrations
MLST: Multilocus sequence type
PCR: Polymerase Chain Reaction
ST: Sequence Type
XDR: Extensively Drug-resistant
MDR: Multi Drug-resistant
