# Supplementary material for: Characterization of novel bacteriophage PSKP16 and its therapeutic potential against β-lactamase and biofilm producer strain of K2-Hypervirulent Klebsiella pneumoniae pneumonia infection in mice model
Source: BMC Microbiol. 2023 Aug 23;23:233. doi: 10.1186/s12866-023-02979-7 (PMC10464470; doi:10.1186/s12866-023-02979-7)
Supplement: Supplementary file 1 — Additional file 1. [file 12866_2023_2979_MOESM1_ESM.docx]

**Figure Supplementary 1** (Figure S1) .The lytic ability of phage PSKP16 against BhvKp strain. Overnight bacterial culture was diluted (10^3^ CFU/mL) and infected with phage PSKP16 at different multiplications of infection (MOIs): 10, 1, 0.1, for 16 h. Bacterial growth was recorded by changes in absorbance (OD600) using an automated multi-mode plate reader. Data displayed in the plot represent the mean ± SD of three independent experiments.

**B**

**A**

**Figure S 2 A,** Assessment of the pH stability of phage PSKP16 at different PH. **B,** Assessment of temperature stability of

Phage PSKP16 at different temperature.

| **Table S1**. Result of CFU assay to quantify viable cells in pre-formed biofilm | | | |
| --- | --- | --- | --- |
| Inoculum | Planktonic Cells (after 24 h) | Biofilm Formatting Cells (after 24 h) | Biofilm Formatting Cells (after 48 h) |
| 1.5×10^7^ CFU/mL | 1.5×10^9^ CFU/mL | 1.5×10^5^ CFU/mL | 1.5×10^6^ CFU/mL |

| **Table S2** Primers used for identification of *K. pneumoniae* genes for encoding AmpC, ESBLs and carbapenemase. | | | |
| --- | --- | --- | --- |
| **References** | **Size** | **Sequence (5' to 3')** | **Target** |
| In study | 383 | F: CGTCTAGTTCTGCTGTCTTG  R: GCGGCGTTATCACTGTATTG | *KPC* |
| In study | 487 | F: GGCGTAGTTGTGCTCTGG  R: TATAGTCACCATTGGCTTCGG | *OXA-48* |
| In study | 232 | F: ATCCACTATCGCCAGCAG  R: CCTCATTCAGTTCCGTTTCC | *SHV* |
| In study | 552 | F: AGGAAGTGTGCCGCTGTATG  R: CTGTCGCCCAATGCTTTACC | *CTX-M* |
| In study | 373 | F: TCGCCGCATACACTATTCTC  R: AACTTTATCCGCCTCCATCC | *TEM-1* |
| In study | 395 | F: ATACCGCCTGGACCGATGAC  R: GAGATTGCCGAGCGACTTGG | *NDM-1* |
| In study | 480 | F: TGTCGCAAGTCCGTTAGC  R: GCAGCACCAGGATAGAAGAG | *VIM* |
| In study | 335 | F: TTAGCGGAGTTAGTTATTGGC  R: TTAGTTACTTGGCTGTGATGG | *IMP* |
| (92) | 520 | F: GCT GCT CAA GGA GCA CAG GAT  R: CAC ATT GAC ATA GGT GTG GTG C | *MOX* |
| (92) | 190 | F: AAC ATG GGG TAT CAG GGA GAT G  R: CAA AGC GCG TAA CCG GAT TGG | *FOX* |
| (92) | 462 | F: TGG CCA GAA CTG ACA GGC AAA  R: TTT CTC CTG AAC GTG GCT GGC | *CIT* |
| (92) | 405 | F: AAC TTT CAC AGG TGT GCT GGG T  R: CCG TAC GCA TAC TGG CTT TGC | *DHA* |
| (92) | 346 | F: AAC AGC CTC AGC AGC CGG TTA  R: TTC GCC GCA ATC ATC CCT AGC | *ACC* |
| (92) | 302 | F: TCG GTA AAG CCG ATG TTG CGG  R: CTT CCA CTG CGG CTG CCA GTT | *EBC* |

| **Table S3** Primer use in PCR for virulent genes and capsular typing | | | |
| --- | --- | --- | --- |
| **gene** | **Primer sequence (5’–3’), F/R** | **Amplicon size (bp)** | **Reference** |
| *ybtS* | F: GACGGAAACAGCACGGTAAA  R: GAGCATAATAAGGCGAAAGA | 242 | (93) |
| *iutA* | F: GGGAAAGGCTTCTCTGCCAT  R: TTATTCGCCACCACGCTCTT | 920 | (93) |
| *rmpA* | F: CATAAGAGTATTGGTTGACAG  R: CTTGCATGAGCCATCTTTCA | 461 | (93) |
| *K2* | F: CAACCATGGTGGTCGATTAG  R: TGGTAGCCATATCCCTTTGG | 531 | (93) |
| *fimH* | F: GCTGCTGCTGGGCTGGTC  R: GGTCGGGAACGGGTAAGAGG | 292 | In study |
| *entB* | F: GCATCGGTGGCGGTGGTC  R: CGGCGAACAAGGTCAACTGG | 439 | In study |
| *mrkD* | F: CTGAGTGAAACGGGATATGC  R: AGCGGTATGGTGATGTAGC | 224 | In study |
| *MagA(k1)* | F: CATTGCCGCTACTACAGGAG  R: AGTGAACGAATTGATGCTTGG | 385 | In study |
| *mrkA* | F: AATGTAGGCGGCGGTCAG  R: CTCTCCACCGATAACGCCA | 351 | In study |
| *wcaG* | F: AGCAACCGATTAGTGAGTCC  R: TCAACGCCAGTGCCTACG | 402 | In study |
| *K1* | F: GTAGGTATTGCAAGCCATGC  R: GCCCAGGTTAATGAATCCGT | 1047 | (94) |
| *K2wzy* | F: GACCCGATATTCATACTTGACAGAG  R: CCTGAAGTAAAATCGTAAATAGATGGC | 641 | (94) |
| *zxK5* | F: TGGTAGTGATGCTCGCGA  R: CCTGAACCCACCCCAATC | 280 | (95) |
| *wzyK20* | F: CGGTGCTACAGTGCATCATT  R: GTTATACGATGCTCAGTCGC | 741 | (95) |
| *wzxK54* | F: CATTAGCTCAGTGGTTGGCT  R: GCTTGACAAACACCATAGCAG | 881 | (95) |
| *wzyK57* | F: CTCAGGGCTAGAAGTGTCAT  R: CACTAACCCAGAAAGTCGAG | 1037 | (95) |

| **Table S4** Primers used for identification of Strain Typing (MLST) of *K. pneumoniae***(96)** | | |
| --- | --- | --- |
| **Gene name** | **Sequences (5’ to 3’ end)** | **Amplicon size** |
| *gapA* | F: TGAAATATGACTCCACTCACGG R:CTTCAGAAGCGGCTTTGATGGCTT | 662 |
|  |  |  |
| *infB* | F: CTCGCTGCTGGACTATATTCG R:CGCTTTCAGCTCAAGAACTTC | 462 |
|  |  |  |
| *mdh* | F: CCCAACTCGCTTCAGGTTCAG R:CCGTTTTTCCCCAGCAGCAG | 756 |
|  |  |  |
| *pgi* | F: GAGAAAAACCTGCCTGTACTGCTGGC R:CGCGCCACGCTTTATAGCGGTTAAT | 718 |
|  |  |  |
| *phoE* | F: ACCTACCGCAACACCGACTTCTTCGG R:TGATCAGAACTGGTAGGTGAT | 602 |
|  |  |  |
| *rpoB* | F: GGCGAAATGGCWGAGAACCA R:GAGTCTTCGAAGTTGTAACC | 1075 |
|  |  |  |
| *wzi* | F: GTGCCGCGAGCGCTTTCTATCTTGGTATTCC R:GAGAGCCACTGGTTCCAGAAYTTSACCGC | 580 |
|  |  |  |
